# Amyloid Beta Oligomers-Induced Ca^2+^ Entry Pathways: Role of Neuronal Networks, NMDA Receptors and Amyloid Channel Formation

**DOI:** 10.3390/biomedicines10051153

**Published:** 2022-05-17

**Authors:** Erica Caballero, Elena Hernando-Pérez, Victor Tapias, María Calvo-Rodríguez, Carlos Villalobos, Lucía Núñez

**Affiliations:** 1 Unidad de Excelencia Instituto de Biología y Genética Molecular (IBGM), Universidad de Valladolid and Consejo Superior de Investigaciones Científicas (CSIC), 47003 Valladolid, Spain; kekaballero@gmail.com (E.C.); elena.hernando@ibgm.uva.es (E.H.-P.); victor.tapias@uva.es (V.T.); maria.calvorodriguez@abbvie.com (M.C.-R.); 2 Departamento de Bioquímica y Biología Molecular y Fisiología, Facultad de Medicina, Universidad de Valladolid, 47005 Valladolid, Spain

**Keywords:** Alzheimer’s disease, amyloid β oligomers, calcium

## Abstract

The molecular basis of amyloid toxicity in Alzheimer’s disease (AD) remains controversial. Amyloid β (Aβ) oligomers promote Ca^2+^ influx, mitochondrial Ca^2+^ overload and apoptosis in hippocampal neurons in vivo and in vitro, but the primary Ca^2+^ entry pathways are unclear. We studied Ca^2+^ entry pathways induced by Aβ oligomers in rat hippocampal and cerebellar neurons. Aβ oligomers induce Ca^2+^ entry in neurons. Ca^2+^ responses to Aβ oligomers are large after synaptic networking and prevented by blockers of synaptic transmission. In contrast, in neurons devoid of synaptic connections, Ca^2+^ responses to Aβ oligomers are small and prevented only by blockers of amyloid channels (NA7) and NMDA receptors (MK801). A combination of NA7 and MK801 nearly abolished Ca^2+^ responses. Non-neuronal cells bearing NMDA receptors showed Ca^2+^ responses to oligomers, whereas cells without NMDA receptors did not exhibit Ca^2+^ responses. The expression of subunits of the NMDA receptor NR1/ NR2A and NR1/NR2B in HEK293 cells lacking endogenous NMDA receptors restored Ca^2+^ responses to NMDA but not to Aβ oligomers. We conclude that Aβ oligomers promote Ca^2+^ entry via amyloid channels and NMDA receptors. This may recruit distant neurons intertwisted by synaptic connections, spreading excitation and recruiting further NMDA receptors and voltage-gated Ca^2+^ channels, leading to excitotoxicity and neuron degeneration in AD.

## 1. Introduction

Alzheimer’s disease (AD), first reported by Prof. A Alzheimer in 1907 [1], is a neurodegenerative disorder characterized by a loss of cognition and memory that leads to dementia and finally to neuronal death, remaining a giant social, sanitary and economic burden more than one century later [2]. In fact, the specific mechanisms of the disease are unknown, which could provide a potential reason why there is a lack of early diagnosis and efficient treatment for AD [3]. The progression of the disease is critically associated with the accumulation of amyloid β (Aβ) peptide species, particularly Aβ_1–42_ [4]. Post-mortem examination of brains from AD patients has shown a pathological accumulation of Aβ in senile plaques together with a deposition of intracellular tau protein in neurofibrillary tangles [5]. Mutations in several genes coding for the amyloid precursor protein (APP) and the secretases presenilin 1 and 2—involved in APP cleavage—are believed to cause familial forms of AD [6]. 

Excessive content of Aβ_1–42_ peptides leads to their aggregation into oligomeric forms, which are harmful to neurons and synapses, particularly in the hippocampus and the cerebral cortex, two brain areas involved in learning and memory. It is believed that small soluble aggregates, or oligomers, are the earliest and most toxic amyloid species and may promote increases in cytosolic Ca^2+^ concentration in neurons [7]. In addition, Aβ_1–42_ oligomers may cause mitochondrial Ca^2+^ overload followed by apoptosis [7]. This mechanism may be critical for neuronal damage in AD, since inhibition of mitochondrial Ca^2+^ overload prevents neuronal cell death [7,8,9]. This mechanism may also explain the neuroprotective effects shown by a number of non-steroidal anti-inflammatory drugs (NSAIDs) [10,11,12]. It is believed that aging in neurons is associated with a loss of intracellular Ca^2+^ homeostasis, including enhanced coupling and Ca^2+^ transfer from the endoplasmic reticulum (ER) into mitochondria, as well as to decreased store-operated Ca^2+^ entry (SOCE), a mechanism involved in mushroom spine stability required for memory consolidation [13,14]. Furthermore, neuron aging increases Ca^2+^ responses and susceptibility to neuronal cell loss associated with glutamate receptor activation during excitotoxicity, toll-like receptor 4 activation induced by lipopolysaccharide (LPS) associated with neuroinflammation and Aβ oligomers associated with AD [11,15,16,17,18,19].

Therefore, increased Ca^2+^ responses to Aβ associated with aging or increased oligomer accumulation and reduced clearance rates could play a pivotal role in AD etiology. However, the primary target involved in Ca^2+^ responses is still controversial. Different channels have been proposed to be targeted by Aβ. Initially, Arispe and others reported that amyloid peptides may integrate into biological membranes to promote the formation of amyloid channels permeable to Ca^2+^ [20]. The generation of amyloid channels can increase cytosolic Ca^2+^ concentration that may vary largely for each cell type. Synthetic peptides, such as NA7 (constructed with the native sequence of Aβ_11–17_), can block the Aβ channel conductance [21]. Simultaneously, other authors reported that Aβ oligomers may interact with specific Ca^2+^ channels to increase the level of Ca^2+^, including the activation of NMDA receptors that are involved in both learning and memory [22,23,24]. Memantine, a non-competitive, low- to moderate-affinity antagonist of NMDA glutamate receptors, is the only FDA-approved drug for the treatment of AD that is not an acetyl cholinesterase inhibitor. In addition, P/Q-type Ca^2+^ channels are high-voltage-operated Ca^2+^ channels contributing to vesicle release at synaptic terminals and are reported to be targeted and modulated by Aβ oligomers [25]. Accordingly, the primary target of amyloid oligomers responsible for increased Ca^2+^ concentration remains controversial. The differences observed may be attributable to several factors, including the use of media to prepare synthetic oligomers containing a significant amount of NMDA receptor agonists [22]. Interestingly, central nervous system neurons in primary culture can develop synchronous oscillations of cytosolic [Ca^2+^] mediated by synaptic transmission among different neurons. This has been studied in detail in cerebellar neurons but may also occur in hippocampal and/or cortical neurons [26]. In this scenario, amyloid oligomers may stimulate a single neuron by a particular mechanism and excite distant neurons by synaptic networking activity, thus recruiting other ion channels. Accordingly, it is important to disentangling the primary Ca^2+^ entry pathway activated by Aβ oligomers. In this study, we used well-established models of primary culture of rat cerebellar or hippocampal neurons, as well as other cell models expressing or lacking NMDA receptors to address this important issue 115 years after the discovery of AD.

## 2. Materials and Methods

### 2.1. Animals and Reagents

Wistar rats from postnatal days 0–1 were purchased from the animal housing facility at the University of Valladolid. All rats were sacrificed in agreement with the ethical standards of the University of Valladolid using protocols approved by the European Convention 123/Council of Europe and Directive 86/609/EEC. Fura-2/AM was obtained from Invitrogen (Barcelona, Spain). Fetal bovine serum (FBS) is from Lonza (Barcelona, Spain). Horse serum, neurobasal medium, HBSS medium, B27, L-glutamine and gentamycin are from Gibco (Barcelona, Spain). The solution containing Papain was acquired from Worthington (Lakewood, NJ, USA). The poly-D-lysine and annexin V are from BD (Madrid, Spain). DNase I was purchased from Sigma (Madrid, Spain). Other reagents and chemicals are from Sigma or Merck. NMDA receptor subunit plasmids were a kind gift of Prof. John J. Woodward from the Medical University of South Carolina (MUSC), Columbia, SC, USA.

### 2.2. Cell Lines

Cell lines were purchased from the ATCC (HT29) or provided by Prof. MT Alonso (HEK293 and Jurkat T cells) from the University of Valladolid, Spain, and Prof. L. Stephen Frawley (GT1 neurons) from MUSC. HEK293 cells [27], HT29 cells [28], Jurkat T cells [29] and GT1-7 cells [30] were cultured as previously reported. Briefly, cells were plated at 5 × 10^3^ cells per well in DMEM supplemented with penicillin/streptomycin (1%), L-glutamine (1%), and fetal bovine serum (10%). Cell lines were cultured in standard conditions at 37 °C and 10% CO_2_. They were subcultured once a week and used at passages 3–10.

### 2.3. Mouse Anterior Pituitary Cells

Primary cultures of mouse anterior pituitary (AP) cells were prepared as previously reported [31]. Mice were sacrificed by cervical dislocation, and the anterior pituitary lobes were removed quickly. Then, the tissue was chopped into small pieces and incubated with Hank´s medium containing trypsin 1 mg/mL at 37 °C for 30 min. The pieces were kindly passed through a fire-polished siliconized Pasteur pipette to disperse the tissue into individual cells. Finally, cells were centrifugated at 200× *g* for 7 min, washed twice with HBSS and counted. An average of 1 × 10^6^ cells per pituitary were obtained. Cell viability was determined by Trypan blue exclusion (~5% cell death).

### 2.4. Primary Rat Cerebellar or Hippocampal Neuron Cultures

Cerebellar granule cells derived from Wistar rat pups (5 days old) sacrificed by decapitation [7]. Granule cells were seeded on 12 mm glass coverslips coated with poly-L-lysine and cultured in DMEM medium containing high glucose, low K^+^ concentrations and supplemented with FBS 10%, horse serum 5% and antibiotics (100 u/mL penicillin and 100 µg/mL streptomycin) for 24 h. The next day, the medium was replaced by Sato´s medium supplemented with horse serum 5% [7]. Cells were then cultured for 2–4 days before experiments. Hippocampal neurons derived from P0 Wistar rat pups as previously published [8]. After removal of the brain, meninges were discarded, and the hippocampus was separated from the cerebral cortex. Hippocampal tissue was then cut into small pieces, transferred to a solution containing papain at 20 μg/mL and incubated 30 min at 37 °C. Next, tissue pieces were washed with Neurobasal medium before dissociating them into individual cells. Finally, hippocampal cells were plated in 12 mm glass coverslips coated with poly-D-lysine at 40 × 10^3^ cells per dish. Cells were then cultured in Neurobasal medium supplemented with 2% B27 and 10% FBS with no replacement of medium [9]. Cells were cultured for 24 h or 7–10 DIV before experiments. 

### 2.5. Preparation of Amyloid β Peptide_1–42_ Oligomers

Aβ_1–42_ oligomers were prepared following a protocol reported elsewhere [9]. First, 1 mg of Aβ_1–42_ was allowed to equilibrate 30 min at room temperature (RT) and solved in 222 µL of ice-cold 1,1,1,3,3,3-hexafluoro-2-propanol (HFIP) at 1 mM final concentration. Three aliquots of the solution were taken and incubated for 2 h at RT for monomerization. Then, the Aβ_1–42_/HFIP solution was concentrated using a SpeedVac centrifuge (800× *g* for 10 min at RT). Peptide film was solved in DMSO. After 10 min sonication, the solution was spared in aliquots and frozen at −20 °C. For oligomerization, the aliquots were thawed in saline external medium containing 145 mM NaCl, 5 mM KCl, 1 mM CaCl_2_, 1 mM MgCl_2_, 10 mM glucose and 10 mM HEPES (pH 7.4) supplemented with cupric, ferric and zinc sulfates (0.15 µM FeSO_4_-7H_2_O, 5.2 nM CuSO_4_-5H_2_O and 0.15 µM ZnSO_4_-7H_2_O) at about 80 μM. Finally, samples were sonicated for 10 min and incubated at 37 °C for 24 h before experimental use. Western blot analysis (data not shown) shows that the preparations contained low molecular weight oligomers up to 15–20 kDa composed of trimers and tetramers and fibrils larger than 250 kDa as reported previously [9].

### 2.6. Fluorescence Imaging of Cytosolic Ca^2+^ Concentration

Cytosolic Ca^2+^ responses to amyloid oligomers in primary cultures of rat cerebellar or hippocampal neurons were recorded as previously published [7,15,16]. Neurons were rinsed in saline external medium and incubated with the calcium probe fura-2/AM at 4 nM for 60 min at RT in the dark. Coverslips containing the cells were placed in the perfusion chamber of a Zeiss Axiovert 100 TV fluorescent microscope and perfused continuously with the pre-warmed saline external medium at 37 °C. Next, cells were epi-illuminated alternately at 340 and 380 nm by means of a filter wheel. Light emitted at 520 nm was recorded every 1–5 s using a Hamamatsu ER camera (Hamamatsu Photonics France, Barcelona, Spain). Pixel by pixel ratios of sequential frames were then captured, and intracellular Ca^2+^ concentrations from individual regions of interest corresponding to single cells were estimated from the ratio of fluorescence emission following excitation at 340 and 380 nm. Individual neurons were selected by their morphology, quite different from glial cells in the brightfield. 

For analysis of Ca^2+^ responses to stimuli, we first calculated the percentage of cells showing clear increases in intracellular [Ca^2+^] after stimuli presentation (% responsive cells) as revealed by the change of the slope of the recording F340/F380 ratio. Second, we also determined the maximum increase in the F340/F380 ratio during the stimulation period from the resting value of intracellular Ca^2+^ just before the addition of the stimuli (Δ Ratio). In the cases in which resting cytosolic Ca^2+^ showed oscillations of intracellular Ca^2+^, the resting value was the lowest Ca^2+^ value during the recording period before addition of stimuli. To analyze the effects of specific antagonists, we normalized calcium responses to values obtained in the absence of antagonist (control). As stimuli may induce a minor change in a small fraction of cells, whereas others may induce a similar or larger increase in most of the cells, we also quantified Ca^2+^ responses to obtain a single parameter corresponding to the product of the fraction of the responsive cells by the maximum increase in Ca^2+^ induced by stimuli relative to the value obtained in cells treated with vehicle (% activation).

Synchronous and spontaneous oscillations of intracellular [Ca^2+^] oscillations in primary cultures of rat hippocampal and cerebellar neurons are quite variable. Accordingly, for the analysis of calcium oscillations, it is straightforward to measure the amplitude and/or frequency of those oscillations. To overcome this issue, we developed and reported previously [32] a parameter termed “Oscillations Index (OI)”, which is computed as the average of all changes in cytosolic [Ca^2+^] (in absolute values) during a given period of time. The OI, therefore, is a parameter influenced by both the amplitude and the frequency of Ca^2+^ oscillations. Cells showing no oscillations (e.g., in the presence of TTX) had OI values lower than 0.05. Cells exhibiting large and/or high frequency Ca^2+^ oscillations had OI values about 20-fold larger. Thus, OI values ranged from 0.05 (no Ca^2+^ oscillations) to 1 (high frequency and/or amplitude Ca^2+^ oscillations). 

### 2.7. Statistics

Cytosolic Ca^2+^ concentrations were expressed as the ratio of F340/F380 [15]. Data are presented as mean ± SEM. Student’s *t*-test was used to compare two independent groups. Two-way ANOVA with Tukey’s post hoc test was used to compare more than two groups. For all tests, *p* < 0.05 was deemed significant.

## 3. Results

### 3.1. Ca^2+^ Entry Pathways Activated by Amyloid β Oligomers in Rat Neurons

**Figure 1:**
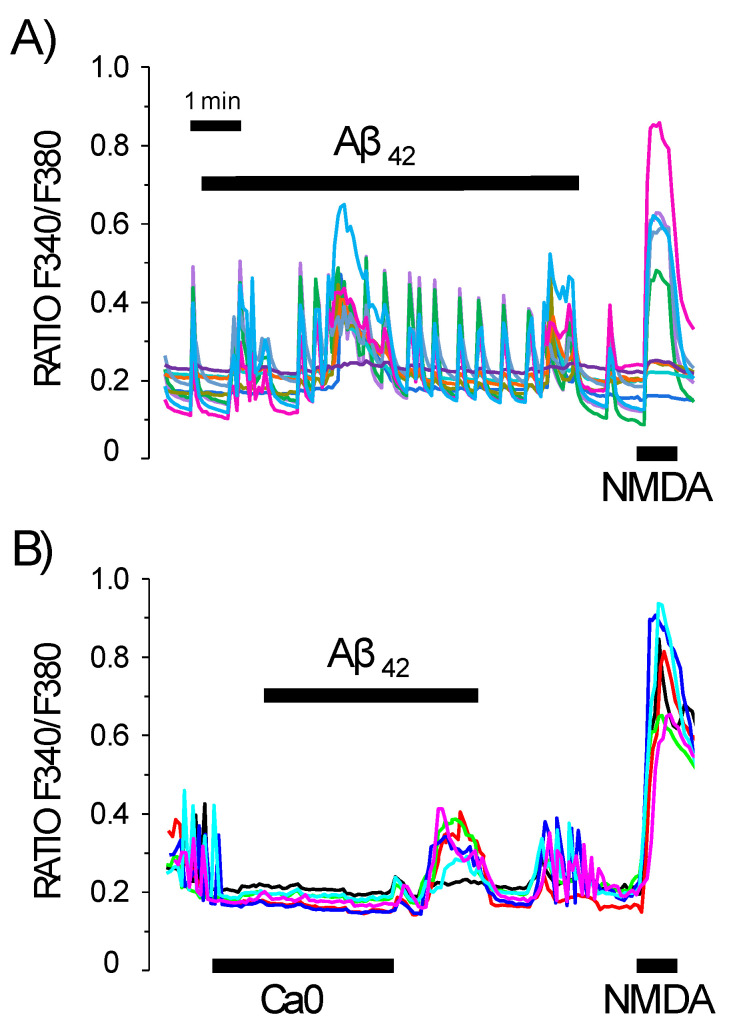
Aß oligomers induce Ca^2+^ entry in rat hippocampal neurons. Rat hippocampal neurons in primary culture (4–5 DIV) were loaded with the Ca^2+^ fluorescent probe fura2/AM and then used for calcium imaging experiments. (**A**) Traces are representative recordings of F340/F380 ratio of individual neurons in the same microscopic field stimulated with amyloid ß_1–42_ oligomers (2 μM) and NMDA (100 μM). Data are representative of 563 cells studied in 46 independent experiments. (**B**) Traces are representative recordings of individual neurons in the same microscopic field stimulated with amyloid ß_1–42_ oligomers (2 μM) in medium lacking extracellular Ca^2+^ when indicated and NMDA (100 μM). Data are representative of 76 cells studied in three independent experiments. Colour lines reflect recordings of individual cells.

Ca^2+^ imaging experiments were carried out in primary rat hippocampal neuronal cultures incubated with Aβ_1–42_ oligomers in a solution containing or lacking 1 mM Ca^2+^. Hippocampal neurons display spontaneous, synchronous oscillations of cytosolic Ca^2+^, which may reflect the synchronous activity of a neuron network (Figure 1). In the presence of exogenous Ca^2+^, Aβ_1–42_ oligomers induced large cytosolic Ca^2+^ increases in most neurons. In contrast, both synchronous Ca^2+^ oscillations and Ca^2+^ responses to amyloid oligomers were abolished in the absence of extracellular Ca^2+^ (Figure 1). Ca^2+^ responses to NMDA were also shown for comparison. These data indicate that Aβ_1–42_ oligomers promote entry of Ca^2+^ through the plasma membrane instead of release of Ca^2+^ from intracellular stores. Similar data were obtained in rat cerebellar neurons (Supplementary Materials, Figure S1).

**Figure 2:**
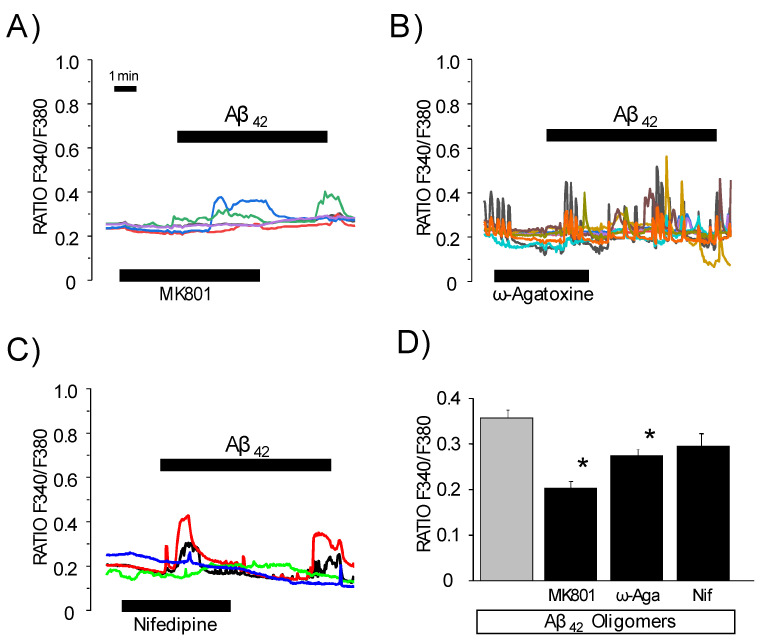
Effects on Ca^2+^ channel antagonists on Ca^2+^ responses induced by Aß oligomers in rat hippocampal neurons. Rat hippocampal neurons in primary culture (3–5 DIV) were loaded with fura2/AM and used for calcium imaging experiments. Traces are representative recordings of F340/F380 ratio of individual neurons in the same microscopic field stimulated with Aß_1–42_ oligomers (2 μM) in the presence of 10 μM NMDA receptor blocker MK801 (**A**), 100 nM P/Q channel antagonist ω-agatoxin (**B**) and 2 μM nifedipine, an L-type channel antagonist (**C**). (**D**) Bars are mean ± SEM values of Δ Ratio from three to five experiments with 25–35 individual cells in each experiment. * *p* < 0.05 vs. control without antagonist. Gray bar corresponds to the effect of oligomers without any antagonist. Colour lines reflect recordings of individual cells.

We sought to identify the potential Ca^2+^ entry pathways recruited by Aβ_1–42_ oligomers. There are several neuronal types of Ca^2+^ channels, such as NMDA receptors or VOCCs. We investigated Ca^2+^ responses to Aβ_1–42_ oligomers in hippocampal neurons in the presence of different channel antagonists, including MK801 (a blocker of NMDA receptor channels), ω-agatoxin (a blocker of P/Q type VOCCs) and nifedipine (a specific blocker of L-type VOCCs). Figure 2 shows representative recordings of Ca^2+^ responses to Aβ_1–42_ oligomers in the presence of the different antagonists. The graphs also exhibits the value of the mean increase in cytosolic Ca^2+^ associated with Aβ_1–42_ oligomers with or without antagonists. We found that Ca^2+^ responses to oligomers were inhibited significantly by MK801 and ω-agatoxin but not by nifedipine. Similar findings were observed in cerebellar or hippocampal neurons (Supplementary Materials, Figure S2). These data suggest that Aβ_1–42_ oligomers may promote Ca^2+^ entry acting on NMDA receptor or P/Q type Ca^2+^ channels but not L-type Ca^2+^ channels. 

### 3.2. Cultured Neurons Develop Neural Networks as Shown by Synchronous Oscillations of Cytosolic Ca^2+^ Concentration That Are Susceptible to Activation by Aβ_1–42_ Oligomers

**Figure 3:**
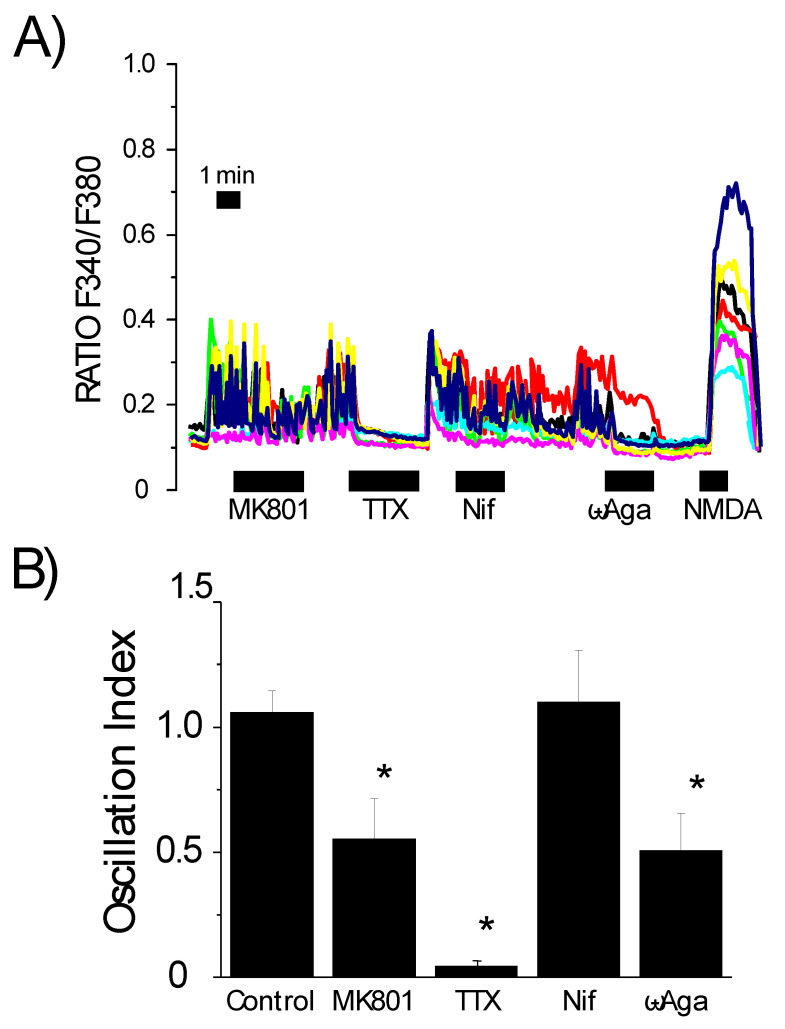
Hippocampal neurons in primary culture display synchronous Ca^2+^ oscillations that are prevented by blockers of synaptic activity. Rat hippocampal neurons in primary culture (7 DIV) were loaded with fura2/AM and used for calcium imaging experiments. (**A**) Traces are representative recordings of the F340/F380 ratio of individual neurons in the same microscopic field in the absence and the presence of several antagonists, including the NMDA receptor blocker MK801 (10 μM), the Na^+^ channel antagonist tetrodotoxin (TTX, 500 nM), the L-type Ca^2+^ channel antagonist nifedipine (Nif, 2 μM) and the P/Q type Ca^2+^ channel antagonist ω-agatoxin (ω-Aga, 100 nM) added before stimulating the cells with NMDA 100 μM. Data correspond to seven individual neurons studied in four independent experiments. (**B**) Bars are mean ± SEM values of the oscillation index obtained in resting conditions during perfusion of the cells with the antagonists shown above. Data are from three to four independent experiments with 43, 28, 15 and 28 cells studied. * *p* < 0.05 vs. control. Colour lines reflect recordings of individual cells.

The main feature of neurons in primary culture is their ability to develop neuronal circuits or networks. Neurons communicate with one another through functional synaptic connections, including glutamatergic synaptic connections. After network formation, the activation of an individual cell might trigger synaptic release of glutamate and other neurotransmitters, which leads to the activation of NMDA receptors and P/Q channels and the firing of synchronous Ca^2+^ oscillations in the entire network. This has already been demonstrated in cerebellar neurons [26] and hippocampal neurons [32]. If this is the case, Aβ_1–42_ oligomers should recruit NMDA receptors and P/Q Ca^2+^ channels indirectly through networking activity rather than direct activation by Aβ oligomers. Thus, we studied the occurrence of synchronous oscillations of cytosolic [Ca^2+^] as surrogate of networking activity in cerebellar and hippocampal neurons and its potential contribution to Ca^2+^ responses induced by Aβ_1–42_ oligomers (Figure 3).

We previously demonstrated that cerebellar neurons in primary culture develop neuronal networks with synaptic connections starting at 6–7 days in vitro (DIV), with clear evidence of the occurrence of synchronous cytosolic Ca^2+^ concentration oscillations [26]. The removal of Mg^2+^ from the extracellular medium (a situation that prevents the Mg^2+^-dependent blockade of NMDA receptor channels) in these cultures causes synchronous cytosolic Ca^2+^ content oscillations. Prior to 6–7 DIV, this experimental maneuver did not induce synchronous oscillations because cultures have not yet developed synaptic connections. In cerebellar neurons, synchronous [Ca^2+^] oscillations were blocked by TTX (96%), an antagonist of voltage-gated Na^+^ channels involved in action potential propagation and network activity. In addition, synchronous oscillations were also blocked by MK801 (90%) and ω-agatoxin (93%) but not by dihydropyridines. These data indicate that synchronous oscillations in cerebellar neurons are dependent on TTX-sensitive, voltage-gated Na^+^ channels, NMDA receptors and P/Q type Ca^2+^ channels. Similar findings were observed in hippocampal neurons (Figure 3). Interestingly, hippocampal neurons often display synchronous cytosolic Ca^2+^ oscillations, even in the presence of extracellular Mg^2+^. As observed in cerebellum cultures, the appearance of synchronous oscillations reflecting networking activity in hippocampal cultures directly depends on culture time. Culture time plays a crucial role in the development of new synapses between dissociated neurons. We observed that, at early time points (1–2 DIV), hippocampal neurons did not show synchronic oscillations, even in the absence of Mg^2+^. However, synchronous oscillations were frequently observed at longer time points (7–8 DIV). Furthermore, synchronic oscillations are promoted under special conditions, such as increased cell plating density. Specifically, we observed primary hippocampal neuronal cultures form neuronal circuits at a density of 70–90 × 10^3^ cells per well. 

To understand the mechanism of synchronization in hippocampal neurons, we assessed the effects of channel antagonists on synchronous activity. Figure 3 shows that synchronic oscillations in hippocampal cultures have a similar behavior compared to cerebellar cultures, since they are also blocked following administration of MK801, TTX or ω-agatoxin. Accordingly, NMDA receptors, P/Q-type Ca^2+^ channels and voltage-gated Na^+^ channels, but not L-type Ca^2+^ channels, are also involved in generating the synchronous activity in hippocampal neurons. Quantitative analysis of Ca^2+^ oscillations was carried out by means of the oscillation index, a parameter that shows elevated values in cells showing high frequency and/or high amplitude of Ca^2+^ oscillations. However, the oscillation index values are low in cells showing no oscillations [31]. We found that MK801 and ω-agatoxin cause a partial inhibition of these oscillations, whereas TTX abolished this activity. Our findings partially differ from previous studies where both MK801 and ω-agatoxin abolished synchronic activity in cerebellar neuronal cultures.

### 3.3. Contribution of the Neuronal Networking Activity to Ca^2+^ Responses Induced by Aβ_1–42_ Oligomers

**Figure 4:**
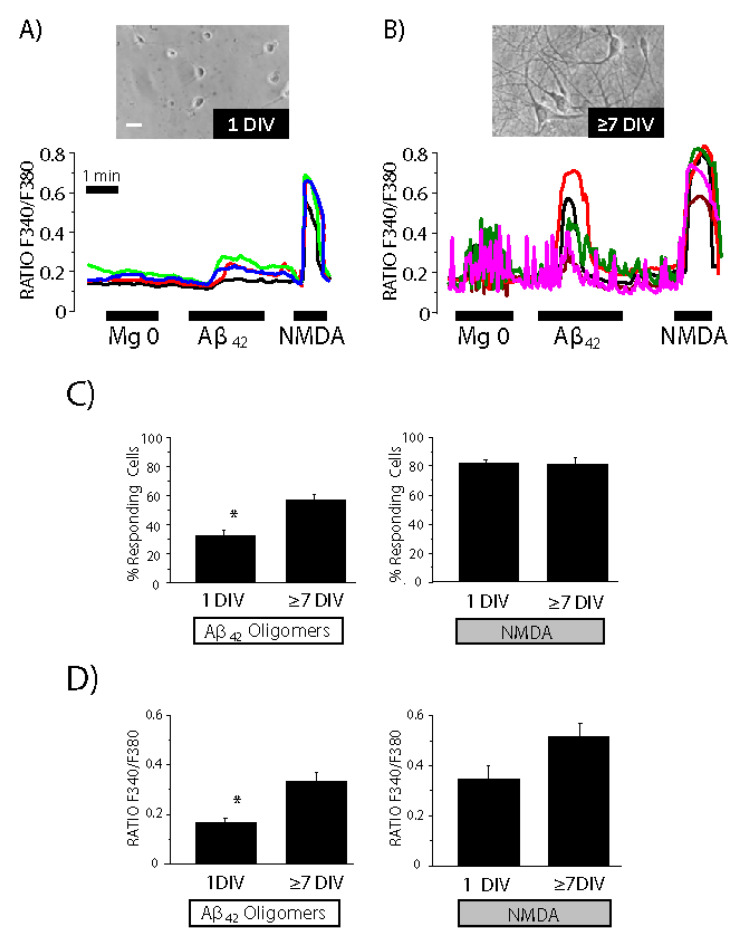
Ca^2+^ responses induced by Aß oligomers are enhanced in cells showing synchronous Ca^2+^ oscillations. Rat hippocampal neurons in primary culture were loaded with fura2/AM and used for calcium imaging experiments. Traces are representative recordings of the F340/F380 ratio of individual neurons cultured for 1 DIV (**A**) or 7 DIV (**B**) stimulated with medium lacking extracellular Mg^2+^, 2 μM Aß_1–42_ oligomers and 100 μM NMDA. Bright-field images representative of hippocampal neurons at 1 and 7 DIV. Recordings correspond to 4 individual cells each panel, representative of 196 and 284 cells studied in 12 and 18 independent experiments, respectively. Bars show the mean ± SEM values of percent (%) of responsive cells to Aβ oligomers and NMDA (**C**) at 1 DIV and 7 DIV. Bars also show mean ± SEM values of Δ Ratio of the Ca^2+^ responses to Aβ oligomers and NMDA at 1 DIV and 7 DIV (**D**). * *p* < 0.05 vs. 7 DIV. Colour lines reflect recordings of individual cells.

To investigate the contribution of network activity to the effects of Aβ_1–42_ oligomers, we examined Aβ oligomer-mediated Ca^2+^ responses before and after network formation. In particular, we studied the effects of oligomers on intracellular Ca^2+^ in cell cultures before circuit formation (1 DIV) and after circuit formation (≥7 DIV). At 1 DIV, Mg^2+^ removal did not cause synchronous oscillations of cytosolic [Ca^2+^] in hippocampal neurons (Figure 4). Nevertheless, neurons displayed Ca^2+^ responses to both Aâ_1–42_ oligomers and NMDA. Similar results were observed in cerebellar cultures (Supplementary Materials, Figure S3). After 7 DIV, hippocampal neurons displayed synchronous cytosolic [Ca^2+^] oscillations, which suggests that there is a networking activity. In these conditions, neurons also displayed an increase in cytosolic Ca^2+^ levels in response to both Aβ_1–42_ oligomers and NMDA (Figure 4). Interestingly, the response to Aβ_1–42_ oligomers was significantly larger at 7 DIV than at 1 DIV, though the response to NMDA was similar. Cerebellar neurons showed similar behavior (Supplementary Materials, Figure S3). These results suggest that networking activity may amplify Ca^2+^ responses induced by Aβ_1–42_ oligomers in both hippocampal and cerebellar cultures. Furthermore, the results indicate that the response to Aβ_1–42_ oligomers can be dissociated from the response to NMDA. This is particularly clear at 1 DIV, where a poor response to oligomers was associated with a large response to NMDA. 

### 3.4. Effects of Channel Antagonists on Ca^2+^ Responses Induced by Aβ_1–42_ Oligomers with or without Networking Activity

**Figure 5:**
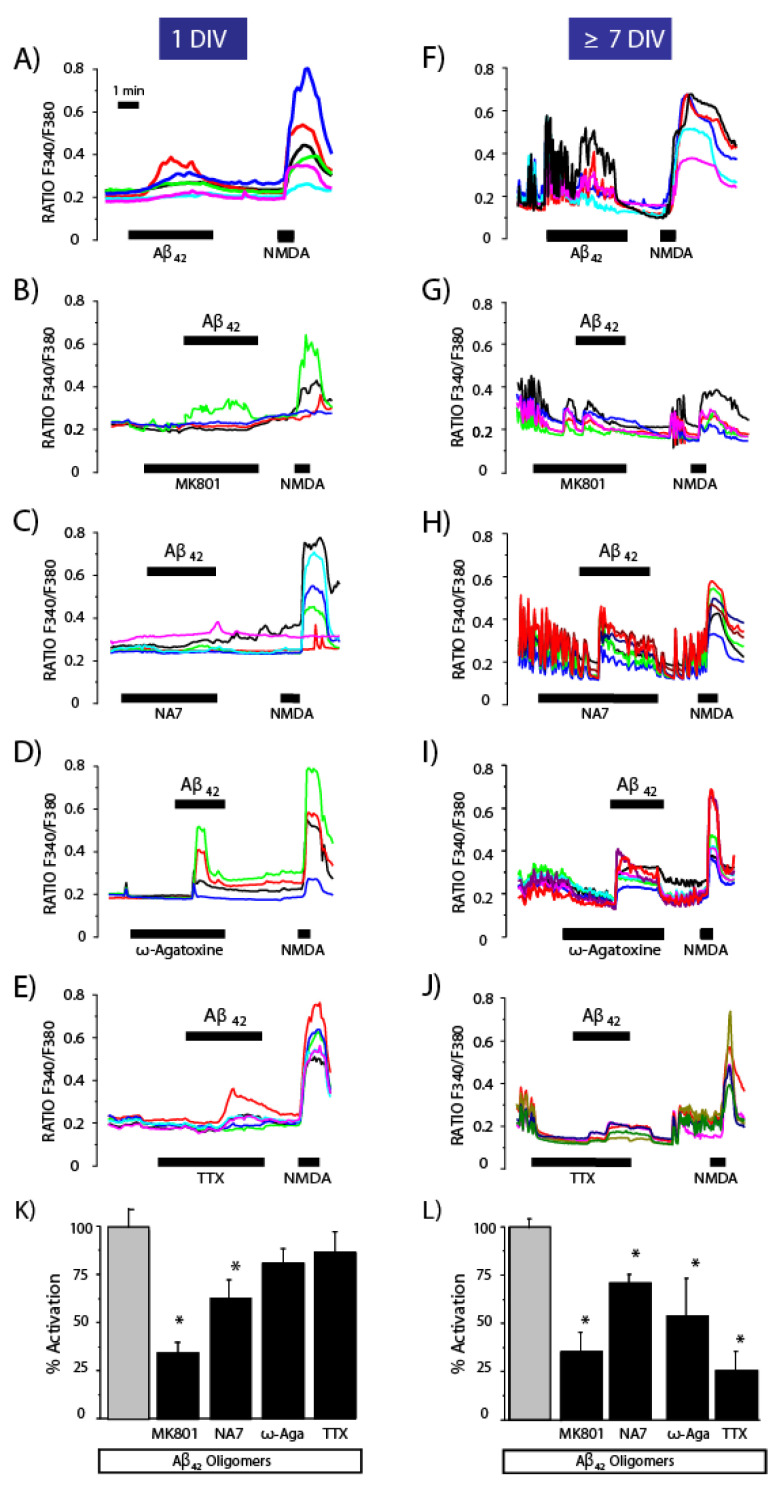
The effects of antagonists on Ca^2+^ responses to oligomers depend on the presence of synchronous Ca^2+^ oscillations. Rat hippocampal neurons cultured either 1 DIV or 7 DIV are loaded with fura2/AM and used for calcium imaging experiments. Traces shown represent typical recordings of the F340/F380 ratio of individual neurons in the same microscopic field stimulated with Aß_1–42_ oligomers (2 μM) in the absence (**A**,**F**) and the presence of the NMDA receptor antagonist 10 μM MK801 (**B**,**G**), 1 μM amyloid channel inhibitor NA7 (**C**,**H**), 100 nM P/Q type Ca^2+^ channel-specific antagonist ω-agatoxin (**D**,**I**) and 500 nM Na^+^ channel antagonist TTX (**E**,**J**). Traces correspond to four to six individual neurons representative of 26201373 cells studied in three to nine independent experiments. (**K**,**L**) Bars represent the mean ± SEM values of the Ca^2+^ response (% activation corresponding to the fraction of responsive cells multiplied by the Δ Ratio) to the oligomers in the absence and the presence of the antagonists at 1 DIV and ≥7 DIV, respectively. * *p* < 0.05 vs. control. Colour lines reflect recordings of individual cells.

We tested whether network activity can modulate the inhibition of Ca^2+^ responses to oligomers induced by Ca^2+^ channel antagonists. In addition to MK801, ω-agatoxin and TTX, we also examined the effects of NA7, a fragment of the Aβ_1–42_ peptide itself (Aβ_11–17_) composed of seven amino acids (EVHHQKL) [20,21] that reportedly block amyloid channels [21]. Primary rat hippocampal neuronal cultures were seeded at a high density (70–90 × 10^3^ cells per well) to favor network activity, and Ca^2+^ responses to Aβ_1–42_ oligomers were tested at 1 and 7 DIV. Figure 5 shows representative Ca^2+^ recordings obtained before (1 DIV) and after (>7 DIV) network formation, respectively. As shown above, Ca^2+^ responses to Aβ_1–42_ oligomers were much larger after network formation. In this scenario, all the agonists tested, including TTX, significantly inhibited Ca^2+^ responses to Aβ_1–42_ oligomers. In contrast, before network formation, only MK801 and NA7 prevented Ca^2+^ responses. Similar results were also obtained in cerebellar neurons in culture (Supplementary Materials, Figure S4). 

These data suggest that Ca^2+^ responses to Aβ_1–42_ oligomers are enhanced by network formation. In the absence of network formation, only MK801 and NA7 blocked Ca^2+^ responses to oligomers, suggesting that NMDA receptors and amyloid channels can be the primary Ca^2+^ entry pathways induced by amyloid oligomers.

**Figure 6:**
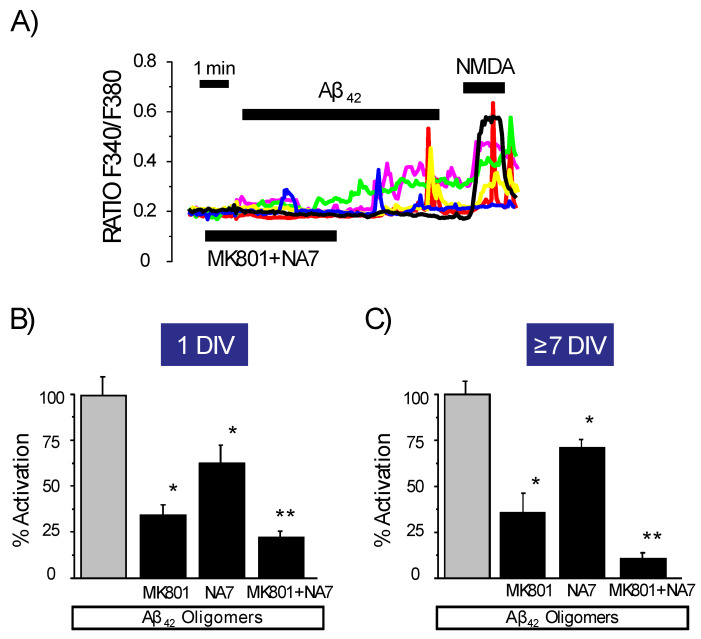
The combination of MK801 and NA7 nearly abolished Ca^2+^ responses to amyloid ß oligomers. Rat hippocampal neurons cultured either 1 DIV or 7 DIV are loaded with fura2/AM and used for calcium imaging experiments. (**A**) Traces are representative recordings of RatioF340/F380 of individual neurons in the same microscopic field stimulated with Aß_1–42_ oligomers (2 μM) in the presence of 10 μM MK801 (NMDA receptor antagonist) and 1 μM NA7 (amyloid channel antagonist) before stimulating them with 100 μM NMDA. Data are representative of 72 cells studied in six independent experiments. Bars represent the mean ± SEM values of % activation (fraction of responsive cells multiplied by the Δ Ratio) of similar experiments in which neurons cultured for 1 DIV (**B**) or 7 DIV (**C**) were stimulated with Aß_1–42_ oligomers in the absence of antagonists, the presence of MK801 or NA7 alone, or added in combination. * *p* < 0.05 vs. control. ** *p* < 0.05 vs. MK801 or NA7 added alone. Colour lines reflect recordings of individual cells.

Interestingly, the combination of NA7 and MK801 (used at a concentration that completely abolishes Ca^2+^ responses to NMDA, Supplementary Materials, Figure S5) inhibited Ca^2+^ responses to Aβ oligomers to a larger extent than each drug used alone (Figure 6). The data suggest that both compounds act on different targets. Consistently with this view, NA7 had no effect on NMDA-induced Ca^2+^ rises or depolarization-induced Ca^2+^ increases, indicating it does not act through NMDA receptors or VOCCs (Supplementary Materials, Figure S5). When added together, NA7 and MK801 nearly abolished the increase in cytosolic Ca^2+^ concentration induced by Aβ_1–42_ oligomers, even in the absence of networking activity at 1 DIV. Similar results were obtained in cerebellar neurons (Supplementary Materials, Figure S6). Thus, NMDA receptors and amyloid channels are most likely the primary Ca^2+^ entry pathways recruited by oligomers.

### 3.5. Expression of NMDA Receptors Is Mandatory for Ca^2+^ Responses to Aβ_1–42_ Oligomers

**Figure 7:**
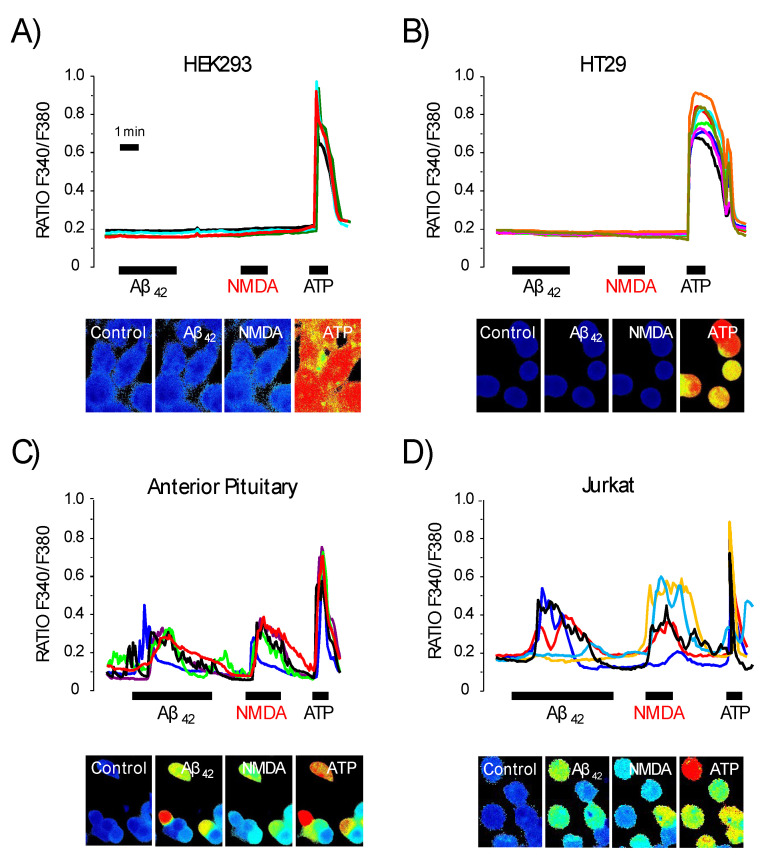
Only cell types expressing functional NMDA receptors also display Ca^2+^ responses to Aß oligomers. Different cell types lacking expression of functional NMDA receptors (HEK293 and HT29 cells) or expressing NMDA receptors (anterior pituitary cells and Jurkat T cells) were cultured, loaded with fura2/AM and used for calcium imaging experiments. Traces are representative recordings of fluorescence F340/F380 ratios of individual HEK293 cells (**A**), HT29 cells (**B**), mouse anterior pituitary cells (**C**) and Jurkat T cells (**D**) stimulated sequentially with Aß_1–42_ oligomers (2 μM), NMDA (100 μM) and ATP (100 μM). Images below recordings correspond to representative pseudo color Ca^2+^ images of each cell type before and after stimulation with each agonist. Notice that dark blue corresponds to low intracellular Ca^2+^ concentration, whereas red denotes high intracellular Ca^2+^ concentration. Data correspond to 4–8 representative cells from 126, 132, 147 and 98 cells studied of each type in six, five, five and four independent experiments, respectively. Colour lines reflect recordings of individual cells.

To address whether NMDA receptors are the primary targets of Aβ_1–42_ oligomers, the effects of oligomers on cytosolic Ca^2+^ in non-neuronal cells bearing or lacking NMDA receptors were assessed. We used HEK293 human embryonic kidney cells and HT29 human colon carcinoma cells that lack expression of NMDA receptors. We also utilized mouse anterior pituitary (AP) cells [33] and human leukemia Jurkat T cells [34] that express endogenously functional NMDA receptors. Functional expression of NMDA receptors was assessed by testing Ca^2+^ responses to NMDA as index. HEK293 and HT29 cells, which have no Ca^2+^ response to NMDA but respond to ATP, did not show Aβ oligomer-induced Ca^2+^ responses (Figure 7). In contrast, Jurkat T and AP cells that showed clear-cut Ca^2+^ responses to both NMDA and ATP, displayed Ca^2+^ responses to Aβ_1–42_ oligomers (Figure 7). 

**Figure 8:**
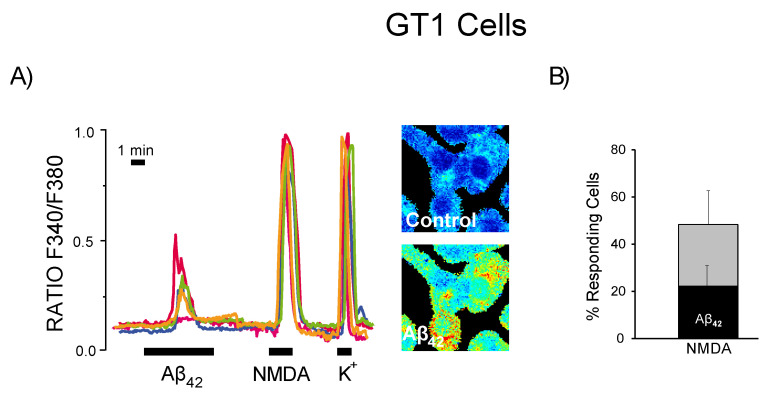
Only GT-1 neurons responsive to NMDA are responsive to amyloid oligomers. GT-1 hypothalamic neurons were loaded with fura2/AM and subjected to calcium imaging. (**A**) Traces are representative recordings of the F340/F380 ratio of individual GT1 neurons stimulated sequentially with Aß_1–42_ oligomers (2 μM), NMDA (100 μM) and depolarizing medium containing a high concentration of K+ (75 mM) to open voltage-gated Ca^2+^ channels. Pictures show pseudo color calcium images GT1 cells before (control) and after perfusion with Aß_1–42_ oligomers. (**B**) Bars show mean ± SEM values of the percent of cells responsive to treatments. Data are representative of 118 cells studied in four independent experiments. Colour lines reflect recordings of individual cells.

Moreover, we tested responses to Aβ_1–42_ oligomers and NMDA in GT1 cells, a hypothalamic neuron cell line also expressing NMDA receptors [35]. We found that only about 50% of the GT1 neurons were responsive to NMDA, and a smaller fraction of about 20% GT1 neurons displayed Ca^2+^ responses to oligomers (Figure 8). Interestingly, all GT1 neurons responsive to Aβ_1–42_ oligomers were also responsive to NMDA. Taken together, these results suggest that NMDA receptor channels are required for amyloid oligomers to induce Ca^2+^ entry in neurons and non-neuronal cells.

### 3.6. Expression of Functional NMDA Receptors Is Not Sufficient for Ca^2+^ Responses Induced by Aβ_1–42_ Oligomers

**Figure 9:**
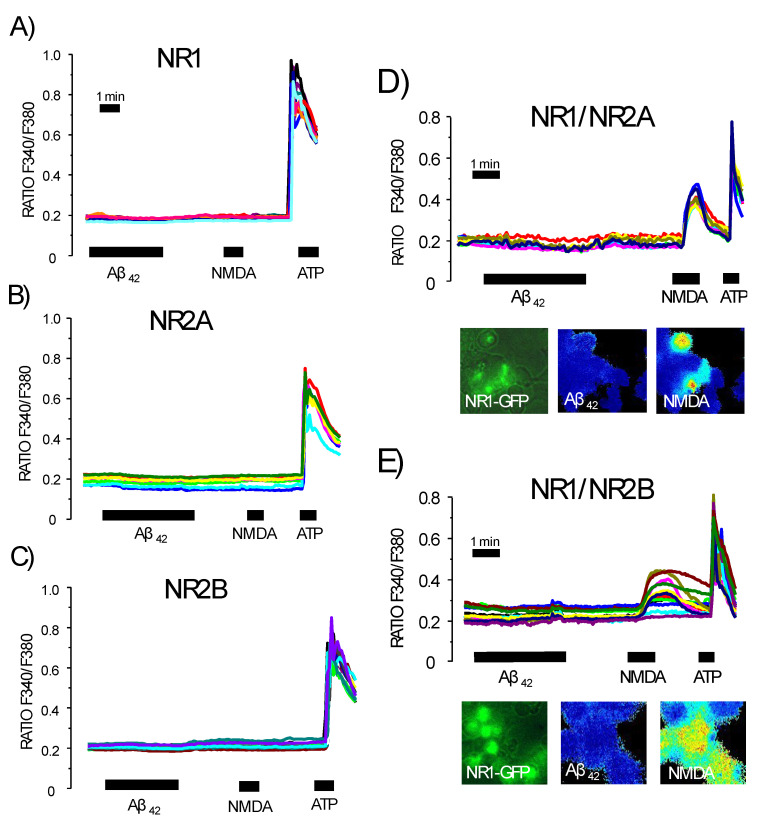
Expression of NMDA receptor subunits in cells lacking NMDA receptors restores Ca^2+^ responses to NMDA but not to oligomers. HEK293 cells lacking endogenous expression of NMDA receptors were transfected with NMDA receptor subunits co-expressing GFP and subjected to calcium imaging. Traces are representative recordings of the F340/F380 ratio corresponding to individual transfected cells with NR1 (**A**), NR2A (**B**), NR2B (**C**) and the combinations NR1/NR2A (**D**) and NR1/NR2B (**E**), stimulated sequentially with Aß_1–42_ oligomers (2 μM), NMDA (100 μM) and ATP (100 μM). Data are representative of 184, 153, 169, 345 and 367 cells studied in four to eight independent experiments for each condition. Pictures show GFP fluorescence images and calcium pseudo color images during stimulation with Aß_1–42_ oligomers and NMDA. Notice that dark blue pseudo color corresponds to low cytosolic Ca^2+^ concentration, whereas red pseudo color denotes high cytosolic Ca^2+^ concentration. Colour lines reflect recordings of individual cells.

We investigated whether exogenous expression of the NMDA receptor in cells lacking endogenous expression of NMDA receptors induced a response to oligomers. HEK293 cells were transfected with different subunits of the NMDA receptor, including NR1, NR2A and NR2B [36]. To be functional, the NMDA receptor requires NR1 subunits combined with NR2A or NR2B. Consistently, we found that the expression of a single subunit, either NR1 or NR2, is not enough for recording Ca^2+^ responses to NMDA in spite of cells being perfectly responsive to ATP (Figure 9). In these transfected cells, Aβ_1–42_ oligomers were also unable to induce Ca^2+^ entry. Moreover, HEK293 cells were co-transfected with different subunits to allow the formation of a complete functional complex. Ca^2+^ responses to NMDA were clearly recorded for the combinations of NR1 with NR2A and NR1 with NR2B. However, in these co-transfected cells, Aβ_1–42_ oligomers did not induce Ca^2+^ entry. Thus, Ca^2+^ responses to NMDA and oligomers can be also dissociated, suggesting that NMDA receptor expression, although necessary, may not be sufficient for enabling Ca^2+^ responses to oligomers. 

## 4. Discussion

Neuronal dysfunction and death are prominent features of the pathogenesis of AD related to changes of unknown origin in the APP metabolism, which leads to excessive levels of the long form of the amyloid peptide Aβ_1–42_ compared with the short Aβ_1–40_ form. Although quantitatively, the changes are not very important, the long form is significantly more hydrophobic and fibrillogenic, which makes it more prone to aggregation. Both forms can form dimers and tetramers, but only Aβ_1–42_ can exist in larger soluble aggregates, collectively referred to as Aβ peptide oligomers. Growing evidence suggests that specific oligomeric species are the most toxic forms of Aβ, which cause synaptotoxicity and neuronal damage. However, the mechanism of Aβ_1–42_ accumulation and aggregation is unknown. A possible explanation is related to an increased peptide synthesis, a defective clearance or both. In familial forms of the disease, excessive levels of Aβ_1–42_ are associated with mutations in the APP protein or in the presenilins involved in γ-secretase activity [6]. Sporadic AD accounts for over 90% of cases and has a later age at onset of 65 years of age. Although the exact cause is not known, evidence indicates that subjects carrying the ε4 allele of apolipoprotein E (APOE) are at increased risk of developing late-onset AD. ApoE4 stabilizes toxic Aβ oligomers and impairs autophagic clearance, thereby promoting its aggregation. Thus, deciphering the molecular mechanisms underlying the Aβ peptide oligomer-induced neurotoxicity is critical for understanding the etiology of the disease and for developing novel therapeutic strategies. 

Work by our lab and others has shown that oligomer-mediated neurotoxicity is related to the effects on intracellular Ca^2+^ homeostasis [7,8,9,10,11,12,13,37,38,39]. We demonstrated that Aβ oligomers promote Ca^2+^ entry followed by mitochondrial Ca^2+^ overload, which leads to neuronal death via apoptosis in both cerebellar and hippocampal neurons in primary culture [7,8]. Multiphoton imaging provided compelling evidence that Aβ oligomers increase Ca^2+^ levels in the cytosol [40] and mitochondria [41] in the brain of living mice, which precedes neuronal death in AD. Nevertheless, the primary Ca^2+^ entry pathways activated by Aβ oligomers remain controversial. In this study, we showed that Aβ oligomers induce Ca^2+^ responses in rat cerebellar and hippocampal neuronal cultures. These responses were specifically observed in neurons but not in glial cells, as previously reported [7,8,9]. Ca^2+^ responses were blocked in the absence of extracellular Ca^2+^, suggesting that they were originated due to a stimulation of Ca^2+^ entry from outside the cell and not by a release from intracellular stores. Other authors stated that Aβ and NMDA receptor activation may cause mitochondrial dysfunction involving ER Ca^2+^ release in cortical neurons [42]. 

The relevant question that immediately arises is whether it would be possible to stop disease progression and neuronal loss by inhibiting Ca^2+^ overload in cells. To answer this question, it is necessary to identify the molecular target(s) and the mechanism(s) by which Aβ peptide oligomers induce Ca^2+^ entry and/or Ca^2+^ mobilization into neurons. Different molecular targets have been proposed, including several Ca^2+^ channels, such as glutamate receptors, especially the NMDA receptor [22,23,24]. However, the medium utilized for oligomerization can contain traces of glutamate receptor agonists [43], thus questioning this hypothesis. Our lab developed a protocol for oligomer preparation lacking glutamate receptor agonists that still promotes Ca^2+^ entry, mitochondrial Ca^2+^ overload and neuronal death in both cerebellar and hippocampal neuronal cultures [9]. We used this preparation here to test the effects of specific antagonists to identify the primary target of Aβ oligomers. Surprisingly, we found that almost all the antagonists tested inhibited Ca^2+^ responses to oligomers. This suggests that oligomers interact and activate multiple Ca^2+^ entry pathways or, alternatively, oligomers induce a single pathway in some neurons, and excitation potentially spreads throughout a network of interconnected neurons. This second possibility is supported by the observation that cerebellar or hippocampal cultures do develop actual neuronal circuits. 

Moreover, synchronous Ca^2+^ oscillations can be observed in resting conditions but are generally activated after removing Mg^2+^, which blocks the NMDA-type receptor in a voltage-dependent manner. Synchronous Ca^2+^ oscillations are blocked following treatment with MK801, P/Q-type Ca^2+^ channel antagonists and TTX. Our results support the mechanism of synchronous Ca^2+^ oscillations in rat hippocampal neurons, which are able to generate networks in primary culture even earlier than cerebellar neurons [32]. Synchronization also occurs in the presence of Mg^2+^, suggesting that these neurons are more excitable than cerebellar cells, and/or they involve other excitatory receptors not inhibited by extracellular Mg^2+^. Hippocampal neurons are also sensitive to MK801, P/Q channel antagonists and TTX but, in contrast to cerebellum, only TTX can block network activity. In addition, MK801 and ω-agatoxin were able to partially inhibit synchronous Ca^2+^ oscillations, thereby suggesting that other types of receptors (in addition to NMDA-type receptors) could play an important role in the synchronization of these cells and the formation of neural circuits. 

Our results showed that primary cultures of cerebellar and hippocampal neurons display synchronous oscillations, which start to be evident after the 3–4 DIV and consistently after 7 DIV. To determine the potential contribution of circuit formation to Ca^2+^ responses to oligomers, we investigated the effects of oligomers on neurons before (1 DIV) and after (7 DIV) circuit formation. Our findings demonstrated that oligomers induce Ca^2+^ responses before and after circuit development. Nevertheless, the number of cells responding to oligomers and the rise in Ca^2+^ concentration were significantly higher at 7 DIV relative to 1 DIV. The data suggest that circuit formation amplifies the response to oligomers. An alternative explanation might be related to a change in the expression of receptors for the oligomers, but our results show that the Ca^2+^ responses to NMDA are similar before and after circuit development. 

Our findings also suggest that circuit formation could amplify the response to oligomers. Consistently, we found that TTX (which inhibits action potential propagation) is able to largely inhibit the effect of oligomers at 7 DIV, though it has no effect at 1 DIV. In addition, MK-801 and ω-agatoxin inhibit the effect of the oligomers and synchronous Ca^2+^ oscillations. In the absence of a neuronal circuit (1 DIV), only MK-801 is capable of consistently preventing the effect of oligomers in the cerebellum and hippocampal neurons. These observations suggests that the NMDA receptor could act not only as an amplifier of the response but might also play an essential role as a direct target.

Oligomers can also target the amyloid channels. It has been proposed that oligomers could insert themselves and form selective pores (channels) in the plasma membrane of some cell types [20,21]. Arispe and colleagues developed a series of peptides capable of inhibiting the activity of amyloid channels [21]. We used NA7, the most effective of these synthetic peptides, to determine the possible involvement of amyloid channel formation in Ca^2+^ entry induced by oligomers. NA7 was able to inhibit oligomer-induced Ca^2+^ entry at 7 DIV, and this effect was independent of the inhibition of voltage-gated Ca^2+^ channels or NMDA receptors. Furthermore, NA7 was able to inhibit Ca^2+^ responses induced by the oligomers even in the absence of a neuronal circuit. Taken together, these findings suggest that oligomers could directly activate NMDA receptors and/or form amyloid channels, as well as excite some hippocampal neurons. This activation could be propagated and amplified through the neural network. In support of this notion, we demonstrated that the combination of NA7 and MK801 inhibits more efficiently Ca^2+^ responses compared to each compound alone. NA7 and MK801 practically blocked the Ca^2+^ response entirely, thus indicating that both act through independent pathways and that NMDA receptors and amyloid channels are primary targets of amyloid oligomers.

Our study confirmed that NMDA receptors are the primary targets of amyloid oligomers. Cell lines bearing NMDA receptors and showing Ca^2+^ responses to NMDA, such as anterior pituitary cells, Jurkat T cells and GT-1 neurons, were also responsive to amyloid oligomers. In contrast, cells without NMDA receptors that consistently lacked Ca^2+^ responses to NMDA were not responsive to amyloid oligomers. These data indicate that NMDA receptors are vital to generate a Ca^2+^ response to oligomers. However, the expression of NMDA receptors may not be sufficient, since the expression of NR1 and either NR2A or NR2B in HEK293 cells lacking endogenous NMDA receptors restored Ca^2+^ responses to NMDA but not to amyloid oligomers. 

In summary, we propose that a primary mechanism induced by oligomers in hippocampal neurons would be the formation of amyloid channels, a process that may require the expression of NMDA-type receptors. There is an additional unidentified component responsible for at least 20% of Ca^2+^ entry induced by the oligomers. The stimulation of Ca^2+^ influx into some neurons developing a neural network would induce activation of all interconnected neurons, depolarization of presynaptic neurons, action potential propagation, activation of voltage-dependent channels, exocytosis of neurotransmitters (mainly glutamate), activation of NMDA-type glutamatergic receptors and synchronous activity of the entire network. Therefore, the NMDA receptor and the amyloid channels seem to be the primary targets in the activation of a lethal Ca^2+^ influx to neurons. The controversial relationship between NMDA-like receptors and amyloid channel formation remains to be further clarified.

## Data Availability

All original data are available upon request.

## Supplementary Materials

The following supporting information can be downloaded at: https://www.mdpi.com/article/10.3390/biomedicines10051153/s1, A single file containing all the Supplementary Figures S1–S6, including the legends of the supplementary, Supplementary Figures Caballero et al.pdf.
