# Supplementary material for: Amyloid Beta Oligomers-Induced Ca2+ Entry Pathways: Role of Neuronal Networks, NMDA Receptors and Amyloid Channel Formation
Source: Biomedicines. 2022 May 17;10(5):1153. doi: 10.3390/biomedicines10051153 (PMC9138537; doi:10.3390/biomedicines10051153)
Supplement: Supplementary file 1 [file biomedicines-10-01153-s001.zip › biomedicines-1685906-supplementary.pdf]

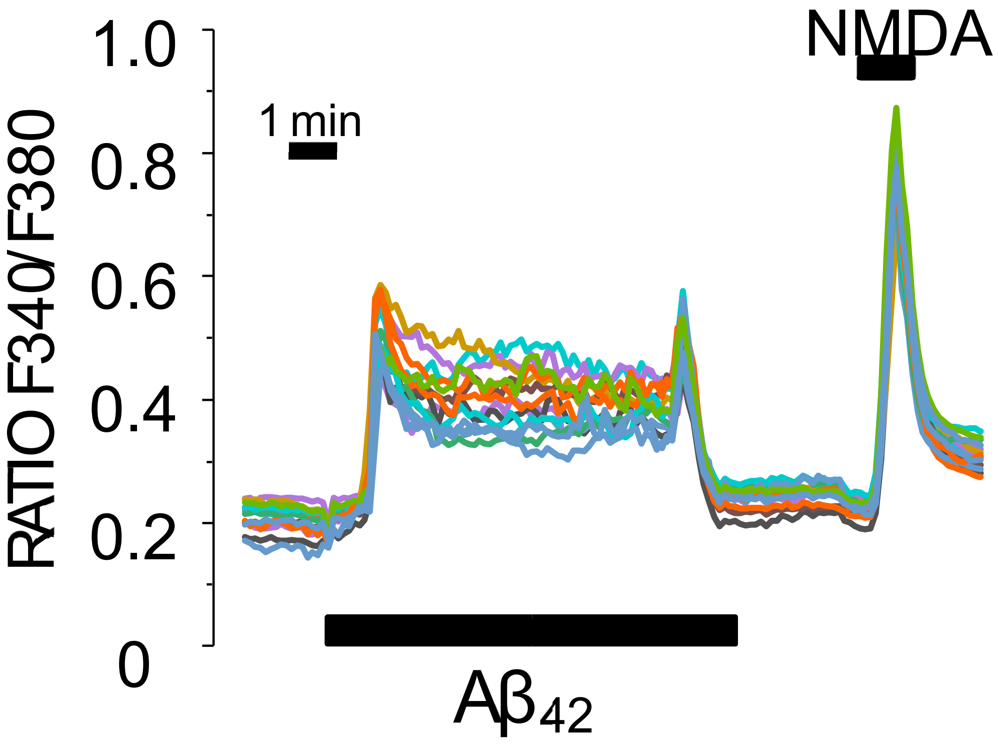

Supplementary Figure S1. Amyloid ß oligomers induce Ca^{2+} responses in rat cerebellar neurons. Rat cerebellar neurons in primary culture were loaded with fura2/AM and subjected to calcium imaging. Traces are representative recordings of Ratio F340/F380 of individual neurons in the same microscopic field stimulated with amyloid $ß_{1-42}$ oligomers (2 μM). Data corresponds to 6 individual neurons and are representative of 5 independent experiments.

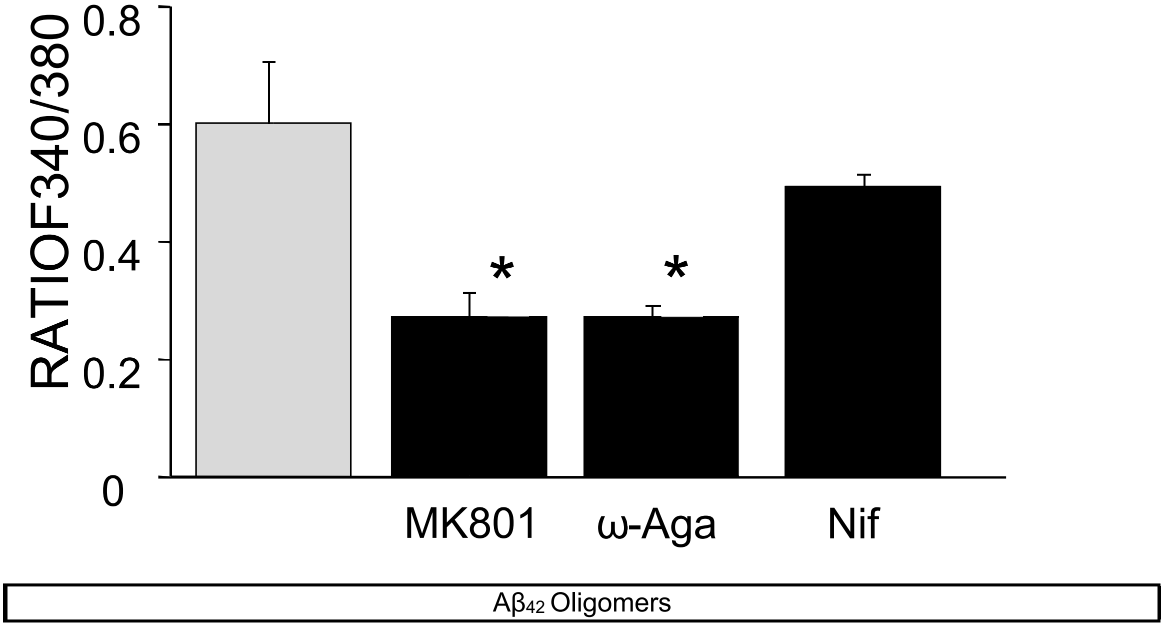

Supplementary Figure S2. Effects on Ca^{2+} channel antagonists on Ca^{2+} responses induced by amyloid ß oligomers in rat cerebellar neurons. Rat cerebellar neurons in primary culture were loaded with fura2/AM and subjected to calcium imaging. Cells were stimulated with amyloid ß oligomers in the absence and the presence of NMDA receptor blocker MK801 10 µM, P/Q type Ca^{2+} channel specific antagonist ω-agatoxin 100 nM and L-type Ca^{2+} channel specific antagonist nifedipine (Nif, 2 µM). Bars are mean ± SEM values of ΔRatio from 3-5 experiments with 40-55 individual cells in each experiment. *$p<0.05$ vs control without antagonist.

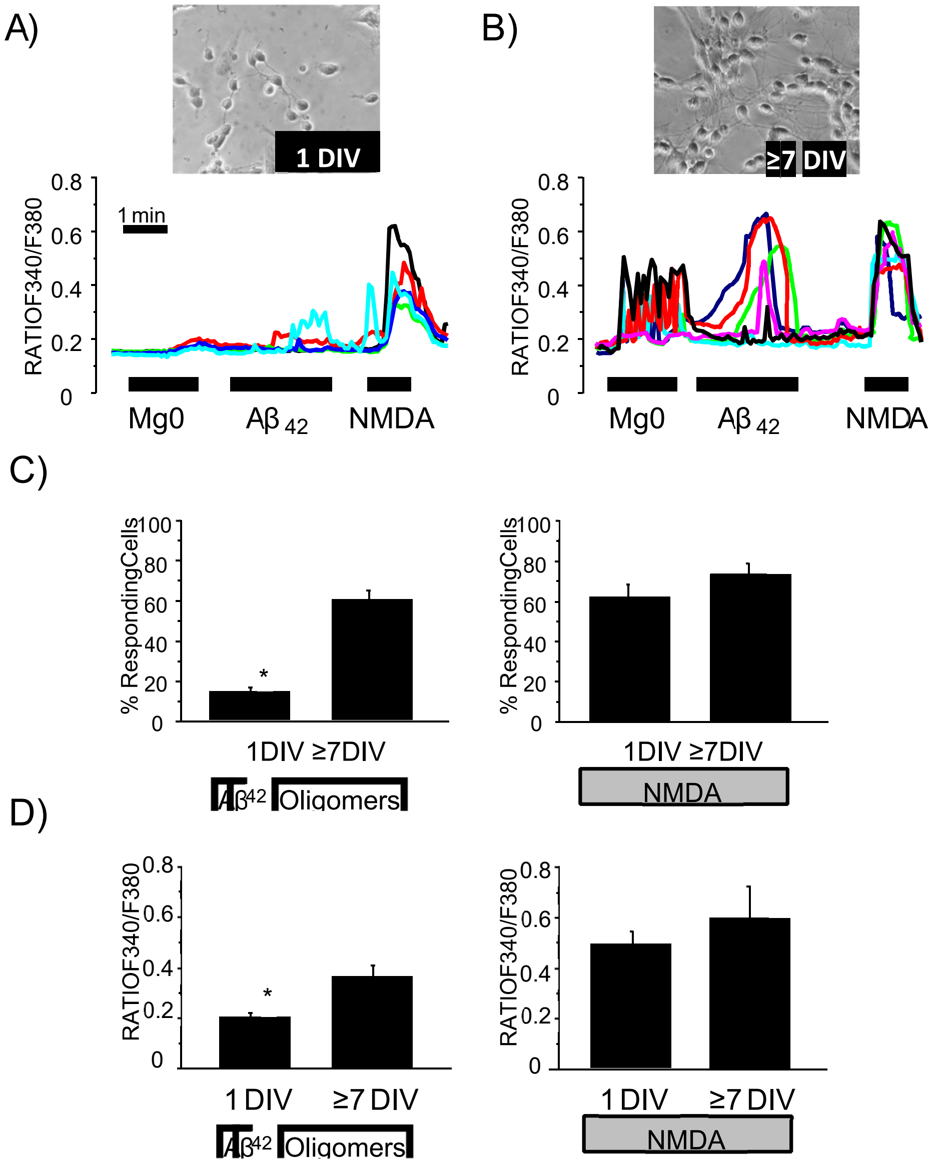

Supplementary Figure S3. Ca^{2+} responses induced by amyloid ß oligomers in cerebellar neurons are enhanced in cells showing synchronous Ca^{2+} oscillations. Rat cerebellar neurons in primary culture were loaded with fura2/AM and subjected to calcium imaging. Traces are representative recordings of Ratio F340/F380 of individual neurons cultured for 1 DIV (A) or 7 DIV (B) stimulated with medium lacking extracellular Mg^{2+} (Mg 0), amyloid $ß_{1-42}$ oligomers ($Aβ_1 42$, 2 µM) and NMDA 100 µM. Pictures are representative bright field images of cerebellar cultures at 1 DIV and 7 DIV. Recordings correspond to 4-6 individual cells representative of 284 and 321 cells studied in 15 and 23 independent experiments, respectively. C. Bars show the mean ± SEM values of percent (%) of responsive cells to Aβ oligomers and NMDA at 1 DIV and 7 DIV. D. Bars also show mean ± SEM values of ΔRatioF340/F380 of the Ca^{2+} responses induced by Aβ oligomers and NMDA at 1 DIV and 7 DIV. *$p<0.05$ vs 7 DIV.

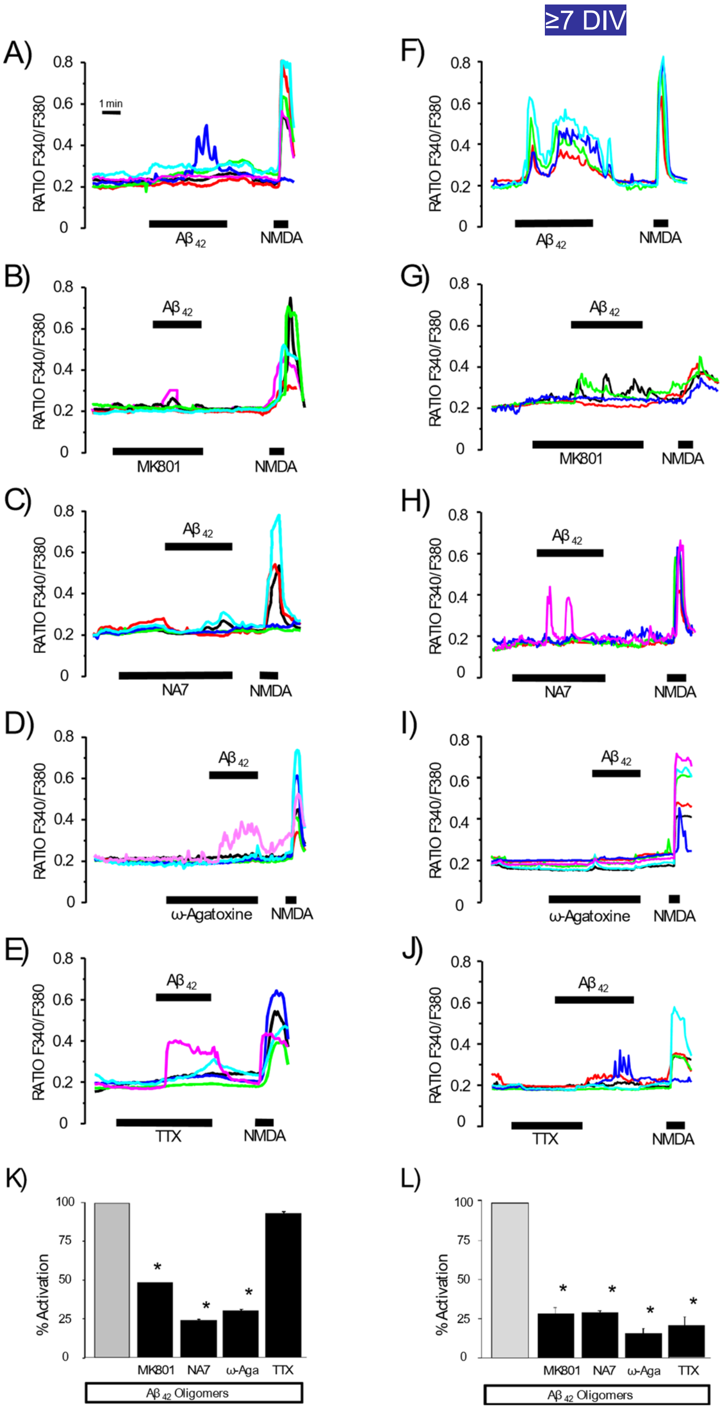

Supplementary Figure S4. The effects of antagonists on Ca^{2+} responses to oligomers in cerebellar neurons depend on the presence of synchronous Ca^{2+} oscillations. Rat cerebellar neurons cultured for 1 DIV or 7 DIV were loaded with fura2/AM and subjected to calcium imaging. Traces are representative recordings of Ratio F340/F380 of individual neurons in the same microscopic field stimulated with $Aß_{1\text{-}42}$ oligomers (2 µM) in the absence (A,F) and the presence of the NMDA receptor antagonist MK801 10 µM (B,G), the amyloid channel inhibitor NA7 1 µM (C,H), the P/Q type Ca^{2+} channel specific antagonist ωagatoxin 100 nM (D,I) and the Na^{+} channel antagonist TTX 500 nM (E,J). Traces correspond to 4-6 individual neurons representative of 160-597 cells studied in 4-11 independent experiments. Bars represent the mean ± SEM values of the Ca^{2+} response (% activation corresponding to the fraction of responsive cells multiplied by the ΔRatio) to the oligomers in the absence and the presence of the different antagonists at 1 DIV (K) and 7 DIV (L). *p<0.05 vs control.

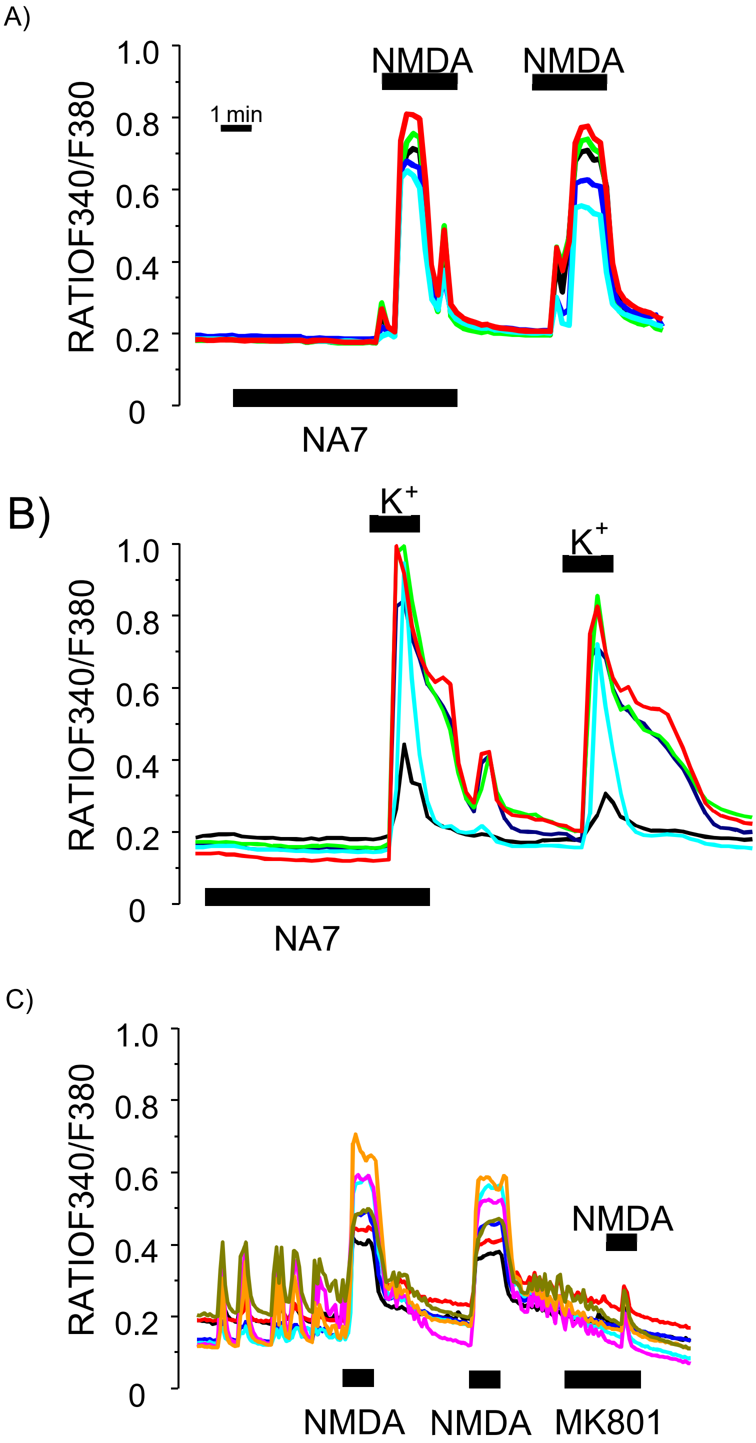

Supplementary Figure S5. NA7 does not inhibit NMDA receptors or voltage-gated Ca^{2+} entry in rat hippocampal neurons. Rat hippocampal neurons were loaded with fura2/AM and subjected to calcium imaging. Traces are representative recordings of Ratio F340/F380 of individual neurons stimulated with NMDA 100 µM (A) and medium containing high concentration of K^+ (B, 75 mM) in the presence and the absence of the amyloid channel blocker NA7 1 µM. C shows representative recordings of Ca^{2+} responses to NMDA in the absence and the presence of MK801 100 µM. Data are representative of 34, 18 and 36 cells studied in at least 3 independent experiments, respectively.

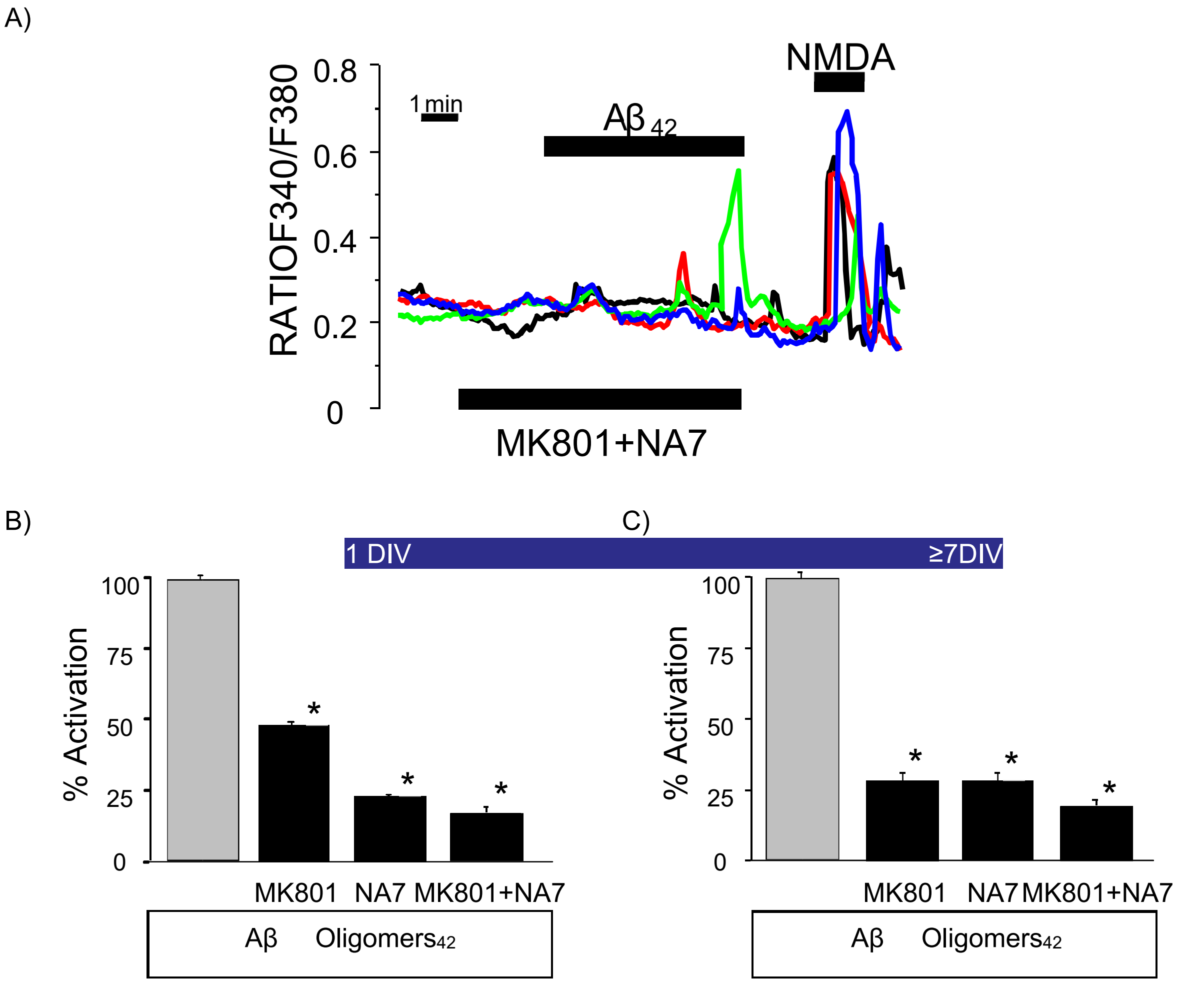

Supplementary Figure S6. The combination of MK801 and NA7 nearly abolish Ca^{2+} responses to amyloid ß oligomers in rat cerebellar neurons. Rat cerebellar neurons cultured for 1 DIV or 7 DIV were loaded with fura2/AM and subjected to calcium imaging. A. Traces are representative recordings of Ratio F340/F380 of individual neurons in the same microscopic field stimulated with $Aß_{1-42}$ oligomers (2 μM) in the presence of the NMDA receptor antagonist MK801 10 μM and the amyloid channel antagonist NA7 1 μM before stimulating them with NMDA 100 μM. Data are representative of 68 cells studied in 5 independent experiments. Bars represent the mean ± SEM values of % activation (fraction of responsive cells multiplied by the ΔRatio) of similar experiments in which neurons cultured for 1 DIV (B) or 7 DIV (C) were stimulated with $Aß_{1-42}$ oligomers in the absence of antagonists or the presence of MK801 and NA7 added alone or in combination.

*p<0.05 vs control. **p<0.05 vs MK801 or NA7 added alone.
